# The Role of a Pacifier Shield: A Unique Perspective on Mandibular Growth and Airway Health: Part 2

**DOI:** 10.3390/children13050643

**Published:** 2026-05-04

**Authors:** Clive Friedman, David A. Tesini, Adithya Kethu

**Affiliations:** 1 Department of Pediatric Dentistry, Schulich School of Medicine and Dentistry, London, ON N6A 5C1, Canada; 2 Private Pediatric Dentistry Practice, London, ON N5X 3W3, Canada; 3 Department of Pediatric Dentistry, Tufts University School of Dental Medicine, Boston, MA 02111, USA; 4 Pediatric/Orthodontic Practice, Natick, MA 01760, USA; 5 Toothprints PC, Hopkinton, MA 01748, USA; adi@toothprints.info

**Keywords:** pacifier, pacifier use, pacifier design, oral health of children, oral habits, growth and development, infant oral health, pediatric airway, pacifier technology, retrognathia

## Abstract

Background: Daily, perhaps for hours at a time, pacifiers are put in baby’s mouths for the primary purpose of soothing. A causal relationship exists between the use of pacifiers and the development of malocclusions and disturbances to the cranio-facial-respiratory complex (CFRC). Finite element analysis (FEA) has enabled us to understand how pacifier bulbs behave under negative pressure and tongue movement in the mouth. It is now realized that the design and size of pacifiers should be based on infant and toddler biometrics of the maxillary palatal width and should contribute to proper oral function and development. Attention has shifted toward the interaction between the pacifier *shield*, the lips, chin and developing tempro-mandibular joint (TMJ). Free mandibular motion is essential to normal TMJ development, essential to maintaining airway space and promoting forward mandibular growth. This is especially relevant in infants with retrognathia, when shield pressure may restrict free movement of the jaw and anterior mandibular advancement. Conclusions: When the pacifier shield interacts with the chin, it impacts the growth and development of the infant mandible and airway physiology. Interference with free movement of the mandible must be made a functional concern in design of the pacifier shield. Technological advances in smartphone photogrammetry now make development of non-invasive diagnostic tools that can advantage the future oral health of children.

## 1. Introduction

Pacifiers have been used for generations, often with the primary purpose of soothing infants and stimulating the suck swallow reflex. Over recent decades, research has identified their relationship with the airway and beneficial effects in reducing the prevalence of sudden infant death syndrome (SIDS), Apparent life-threatening event (ALTE), and a Brief Resolved Unexplained Event (BRUE). Recent studies have examined the causal relationship between pacifiers, the craniofacial respiratory complex (CFRC), and dental malocclusions. The use of facial anthropometrics (biometrics) in baby product design and use has garnered the attentions of engineers, dentists, pediatric providers and the manufacturers of pacifiers. It is no longer acceptable to recommend infant products such as pacifiers, teethers, baby bottles or sippy cups without the knowledge of how harm from improper design or sizing can adversely affect oral development [1].

**Figure 1:**
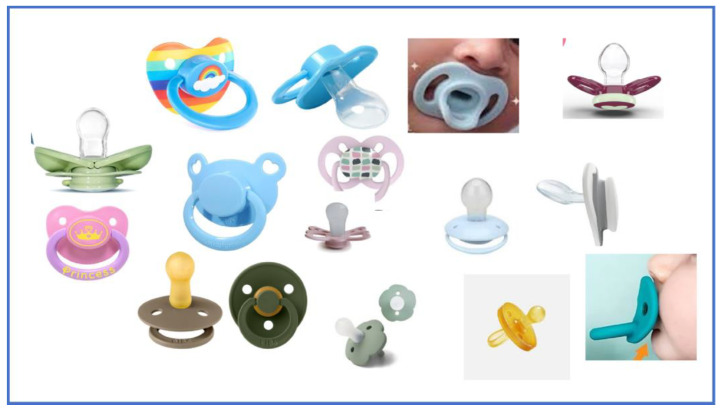
Pacifier Shields in the current marketplace are primarily designed for Consumer Product Safety Commission Standards (CPSC).

Traditional pacifiers consist of two main parts, the bulb and the shield (Figure 1). The pacifier shield has been largely overlooked, and the design has historically been guided by Consumer Product Safety Commission (CPSC) safety standards, particularly those addressing choking risk. These design features—aside from changes in materials, colors, and fashion—have remained largely unchanged over the past six decades. Recently, attention has focused on the functional interaction between the pacifier shield, the lips, the cheeks, and mandible. Pacifier shields should now be designed with awareness of their functionality.

Free mandibular motion is essential to TMJ development, essential to maintaining airway space and promoting forward mandibular growth. This is especially relevant in infants with retrognathia or micrognathia, when shield pressure may restrict anterior mandibular development. Negative pressure generated during sucking draws the shield toward the infant’s face, and the magnitude of this force varies with shield shape, bulb design, bulb fit within the palate, and individual facial anthropometry. These restrictive forces occur during a period of rapid growth of the temporomandibular joint (TMJ) and transitional changes in sucking patterns. The importance of recognizing the retrognathic grower at the time of birth, through use of non-invasive smartphone technologies, helps us to understand potential harmful effects that a pacifier shield can have on infants with retrognathic jaw tendencies. This must be considered when recommending pacifiers to infants and toddlers.

This is the first concept paper to highlight the functional importance of the pacifier shield, and its design, as it relates to growth and development of the infant mandible, airway physiology, and need for the infant to have free movement of their mandible for favorable CFRC development. The development of the concept is based on the functional development of the mandible and the mechanism of pacifier action derived from the peer reviewed literature presented.

## 2. The Anatomic and Functional Importance in Development of the Mandibular Complex

Mandibular growth is most rapid in the first six months of life, with a progressive decline in growth velocity thereafter [2,3,4]. In detail, mandibular size increased 18.2 mm to 34.7 mm between 0.4 and 5 years of age (90.7%) [2]. These rapid changes correspond to transitions in sucking mechanics and tongue peristalsis during early infancy [5]. Masticatory muscle development determines the dynamic development of the TMJ and face through masticatory functioning of posterior occlusion with the functioning of primary first molar around 16 months of age [6,7].

The development of the temporomandibular joint (TMJ) relies on the freedom of mandibular motion during early infancy. The functional adaptation of the TMJ—generated through activities such as chewing, breathing, posture, habitual movements, and even external pressure… produces some of the highest peak loads in the body, supporting both chondrogenesis and fibrocartilage mineralization. Notably, loading of the mandibular condyle is most effective when the condyle is in a protrusive position, particularly during suckling. Suckling acts as a protrusive bite force, which is essential for significant and rapid growth of the TMJ articular eminence [8].

Research indicates that breastfeeding promotes greater mandibular advancement (2.4 mm) compared to bottle feeding (1.6 mm) as infants exhibit less mandibular movement during bottle feeding [9,10]. The compressible nature of breast tissue facilitates natural mandibular movement whereas, a flat rigid plastic pacifier shield, used during non-nutritive sucking (NNS), may restrict this movement.

Transitional changes in sucking mechanics, such as tongue peristalsis, occur within the first few months after birth [5]. Therefore, it is crucial to monitor infants for signs of limited mandibular movement, as this may impact TMJ development and overall airway health.

Early animal studies demonstrated that even food consistency affects condylar morphology and histology [11]. Animal transplantation studies where rat condyles were transplanted into the metacarpal epiphyses further confirmed that condylar growth (both chondrogenesis and osteogenesis) is strongly influenced by its functional environment [12,13].

### Significant Literature into Mandibular Mobility, Mandibular Position, and Airway Obstruction

Dr. Shirley Tonkin, a pioneering researcher in SIDS, described the exceptional mobility of the infant mandible [14].

The TMJ in early infancy lies in a horizontal plane.

- Its shallow articulation allows significant posterior displacement.
- Posterior mandibular movement carries the tongue upward and backward, potentially obstructing the airway when the soft palate contacts the posterior pharyngeal wall.
- Movement of the jaw has important effects on oxygenation.

More recent findings suggest that even mild pressure on the infant mandible may compromise the airway patency [15,16]. This is a concern directly relevant to retrusive forces against the mandible [17] which may also be caused by a pacifier shield.

The position of the developing mandible is a critical factor in airway health, with research linking abnormal mandibular growth or positioning to increased risks of conditions such as SIDS, ALTE, BRUE and Obstructive Sleep apnea (OSA) [18,19]. Although micrognathia refers to mandibular size and retrognathia refers to posterior mandibular position clinically, these terms are often used interchangeably. Both conditions may impair forward tongue posture, resulting in glossoptosis and compromised airway patency [20,21,22]. Clinical neonatal micrognathia (syndromic and non-syndromic) is associated with smaller upper airways and linked to obstructive airways particularly in the first six months of life [22]. Even restrictive pressure on the jaw by the chest during head flexion is associated with narrowing of the upper airway [16,23]. Increased airway obstruction risk correlates with more retrusive mandibular indices [24].

The position of the mandible stabilizes the pharynx and affects the functioning of the velo-pharyngeal mechanism (a dynamic complex separating the oral and nasal cavities during speech) and thus helps prevent airway collapse during sleep [16,18]. The pacifier does provide suction for pharyngeal airway stabilization in the pacifier/SIDS connection [25], but the soft/compressive breast encourages protrusive movement of the mandible better than a restrictive pacifier shield.

Flemming found no difference in SIDS risk between control infants and those who routinely used pacifiers [26], Cozzi however, reported that pacifier use during sleep can improve the maintenance of patent airways and ensure adequate oral airflow in healthy infants. This favored airflow around the pacifier, between the tongue and the palate, and breathing through the mouth serves to sustain an oral airway in the absence of nasal breathing [27]. Others have also found pacifiers to improve the respiratory capacity of children [19,23].

Levrini 2021 reported variations in peripheral capillary oxygen saturation (SpO_2_) during wake time in different positions, during pacifier use. They found a small increase in peripheral oxygen saturation (SpO_2_) percentage in a 0-degree supine position rather than a 30 degree sitting up position [28].

By viewing oxygen saturation data and tongue and jaw position, with the respiratory effect, it reaffirms the importance of mandibular dynamics. Free mandibular movement is needed to maintain upper airway patency [27]. In fact, Maritinot [29] showed that mandibular function is best quantified evidence, via parameters of mandibular jaw movement (MJM) rather than the mere position of the mandible alone. These parameters of MJM acting as “signals” are sent when the neuromuscular engagement of jaw muscles start. These MJM signals strongly correlate (Pearson’s r = 0.84) as the clear physiological marker of respiratory effort rather than oxygen saturation or jaw position alone [30].

Other airway protective mechanisms attributed to pacifier use, such as arousal from sleep if an airway is compromised, have been proposed [31,32]. One such theory, referred to as “bulky handle,” hypothesizes that the pacifier acts as a protective barrier between the infant’s face and bedding [33]. Other risk factors include sleep position, smoking, co-bedding in addition to increased BP and heart rate variability with pacifier use [34,35]. Hanzer however, found that pacifier use does not alter the frequency or duration of spontaneous arousals in sleeping infants [36].

As sleep time use of pacifiers was found to be less than 15 min, parents question if pacifiers continue a preventive effect after they fall out of the infant’s mouth. Cozzi demonstrated that term infants are always able to switch to oral breathing after prolonged nasal occlusion [27].

Any of these protective theories would be difficult to defend if free mandibular movement is inhibited by any force that restricts free movement of the mandible to open or maintain the oral airway. If the mandible is restricted in its forward movement, in any way, it may contradict the theories on the protective effect of pacifier during sleep [37]. Since study results supporting pacifier use are inconclusive and have been challenged, the importance that the pacifier shield allows for free mandibular movement must be considered.

## 3. Improving Shield Design

The physiological and anatomic development of the CFRC (cranio-facial respiratory complex) is important in babies and toddlers and can be impacted by the size and design of the pacifiers they use [1]. Often overlooked, the pacifier shield receives little attention, even as it is functionally seen to restrict the mandible when an infant is sucking on the bulb. The infant’s tongue and facial muscles create a vacuum, causing the outside atmospheric pressure to push the pacifier into the mouth. The infant’s suck creates significant intra-oral negative pressure during NNS of approximately 40 to 230 mbar below atmospheric pressure [38,39,40]. Lindner found that the force of the lips against the canine during dummy sucking was 54 g/cm^2^, with a maximum mean value of intraoral pressure of 260 mbar [41].

**Figure 2:**
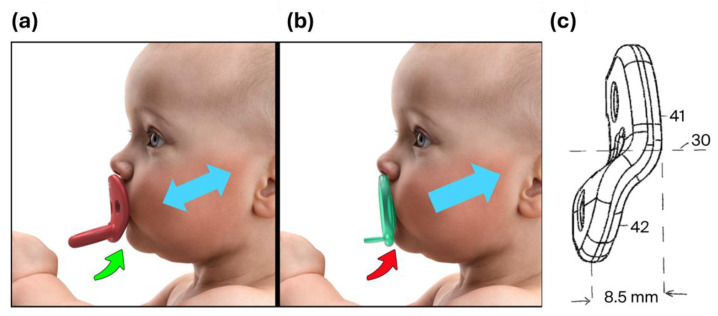
Illustrations showing pacifier shield which is, (**a**) away from the chin during sucking. Free Protrusive and retrusive movement. Notice how the bottom of the shield curves away from the chin, whereas (**b**) the flat restrictive shield entraps the infant mandible in a retruded position. The labeled Inventive Shield (**c**) (30), Centerline of shield (41), main plane of shield (42), inferior portion of shield “off-set” from the main shield plane by 8.5 mm.

Restrictive shields—particularly those with vertically flat contours pressing tightly against the lips—can inhibit mandibular motion by exerting compressive forces during sucking. This mechanical “suck-back” can trap the mandible, impeding normal forward growth. Asano 1986 studied retractive forces on the growing rat mandible and found that there was in fact no catch-up growth after removal of the force [17,42]. This homogeneity in structure and growth processes of the mandibles of mammals, support our concepts that restrictive forces, of either a shield or occlusion, can interfere in the control of TMJ condylar cartilage development. However, when pacifier shields are designed to allow free unobstructed mandibular movement, they may offer protective effects on TMJ and airway development [20]. Figure 2c These animal studies are useful and present valuable insight for future human infant studies on restrictive forces on mandibular motion.

Quantitative research specifying the hourly frequency of pacifier use per day is limited. Parents commonly describe usage patterns with non-specific terms such as “all day long,” “only at night,” “only during the day,” “limited,” “intermittent,” “less intense,” “more intense,” or “exclusively for sleep.” In a recent study, surveying pacifier use and vocabulary, they reported of up to an average of 8 h. of daytime use when child was between 12 and 14 months. Further, the greatest amount of daily use was early in life [43]. In comparison, the relatively short duration of pacifier use during sleep time (78% of babies sucked for less than 15 min), brings into question the etiology of the SIDS/Pacifier connection [37]. We would ask if this short period of sucking time (before the pacifier falls out), actually benefits the renewal of free mandibular movement in the absence of shield interference.

Although the shield transmits these functional sucking forces against the face, cheeks and chin, there have been no studies on the effects of the shield on CFRC development.

Stüdeli 2014 presented ergonomic recommendations for the shield as a ‘functional element’ by recognizing that shield forces against the face should not affect the child’s nose or chin [44]. Pressure points on the chin in rest position and normal range during motion must be avoided [44] Shield impact against the face and chin is dependent on several factors such as shield size, facial type, facial anthropometry, bulb to shield angle, and bulb design. When consideration is given to the facial geometry and convexity of the facial profile, planning for retrognathic growers can be part of the diagnosis and treatment and presented to the parents (Table 1).

Other factors influencing the impact of pacifier shields include the shield’s shape, (such as round, butterfly, or anatomic), the extent of contact with the lips and surrounding facial area, and any retrusive pressure exerted against the chin and mandible. Additionally, variations in the facial type, whether straight, convex or concave—may alter how the shield applies pressure to the jaw during sucking, potentially contributing to a narrowing of the upper airway [16].

Recent advances in facial biometrics have highlighted the importance of selecting a pacifier bulb that fits the palate appropriately, as proper sizing can help support the transverse palatal dimension and prevent adverse effects on the developing craniofacial respiratory complex (CFRC) [1]. While Levrini 2025 study described various bulb characteristics, it did not address how shield design might affect mandibular development or airway patency [45]. It is now essential to apply these biometric principles not only to the bulb design but also to the shield itself.

Sistenich (2022) provided a detailed description of the spatial orientation of the pacifier bulb within the palatal vault [46]. By using anthropometric measurements and referencing Camper’s line, he considered factors such as the intraoral position of the pacifier nipple, the placement of the lip shield on facial soft tissues, lip thickness and shield geometry. The vertical extent of the seated nipple into the palate will affect shield angulation and contact with the chin. Both Studeli and Sistenich’s previous work are precursors to recognizing the functionality of a pacifier shield [44,46].

**Figure 3:**
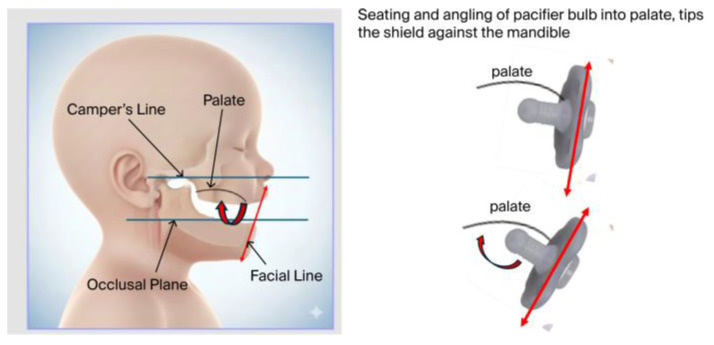
Illustrations showing pacifier bulb as it tips upward to contact the top of the palate during sucking. This shows the resulting effect of the bulb angle and the rotating of the shield plane angle into the chin.

Furthermore, the design of the bulb, the angle between the bulb and shield, the inclination of the bulb tip, and the overall fit of the pacifier must all be considered. Intraoral pressure and the peristaltic action of the tongue push or tip the bulb upward to reach the depth of the palatal vault. As this occurs, the lower part of the shield may tilt backward (retrusive) against the mandible, potentially restricting mandibular movement (see Figure 3; and Supplemental Material Videos).

An inventive “offset shield” (Figure 2c) may allow free movement of the mandible during sucking (Figure 2b). Whether the shield is made of silicone, plastic supported silicone, latex or plastic, an “offset” (in this example 8.5 mm) can be designed into the shield. This proposes to allow for a full range of mandibular motion, which will not interfere with mandibular position and growth of the TMJ. Future studies need to be done on this offset shield design for each brand and infant facial types. It may provide important insights into the hypothesis of the SIDS-pacifier protective benefit. Baby product development companies must be willing to participate in research and education on new pacifier shield designs, so parents and providers will better understand the mechanics and functional impacts of the pacifier shield recommended for their babies.

## 4. Advancing Technology for Orthognathic Screening

Use of advancing smartphone imaging technologies are facilitating development of simple tools for clinical documentation and predictability of mandibular posture. Challenges in early diagnosis of maxillary-mandibular disharmony largely stem from the current avoidance of invasive radiographic technologies during the first years of life. It is largely subjective, *often relying on a clinician’s “clinical impression”*. While several postnatal diagnostic techniques exist (anthropometry, 2D cephalometry, stereophotogrammetry, MRI/CAT), parents and clinicians avoid invasive radiographic imaging techniques. Recognition of mandibular posture in the non -syndromic patient can be particularly difficult and reference norms for mandibular dimensions across age, sex, and ethnicity remain insufficient [3].

Prior research shows that profile photographs are a reliable method for evaluating the soft-tissue profile characteristics, as they correlate well to that obtained by cephalometric images [47,48]. Photography is a cost-effective and reliable method for soft-tissue landmark identification and allows recording images of sufficient quantity for analysis [49,50]. Limitations owing to head positioning, lack of depth perception, and magnification errors must be considered in anthropometric point location [51].

Significant advancements are being made in mobile imaging technology and three-dimensional applications [52]. Modern photogrammetric approaches leverage the principles of stereopsis to estimate depth and enable non-invasive prediction of early indicators of micrognathia and mandibular retrusion.

A novel, non-invasive method for early screening of the infant’s mandibular position has been developed in the *Gnathic-Click™* smartphone application (Toothprints Inc., Hopkinton, MA, USA) This screening tool is designed to augment clinical assessment and support transformative learning in healthcare. To this end, prompted clinical parameters are identified for the clinician, and the use of a lateral facial diagnostic image is used to define the anthropometric AP facial relationship (Table 1).

**Figure 4:**
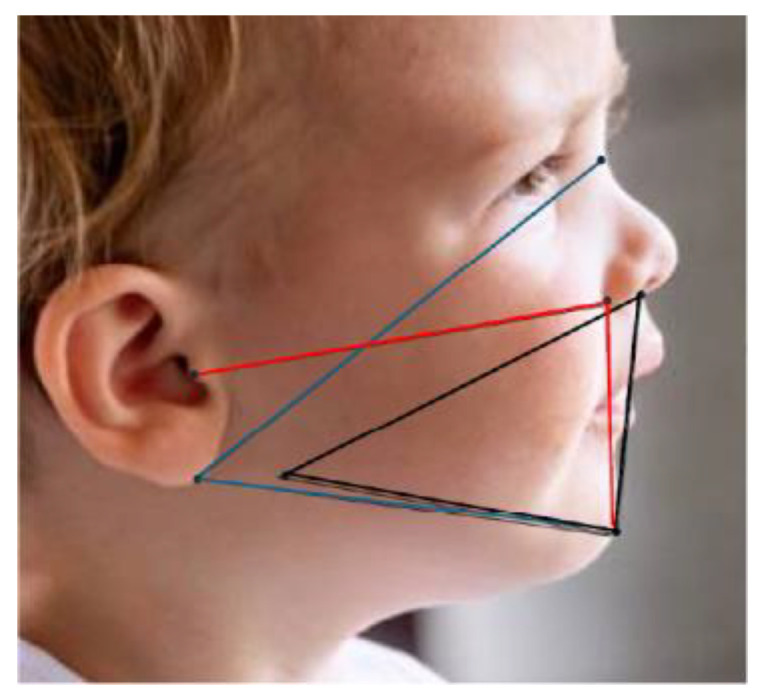
Tracings using published facial anthropometric indices which can be used for developing Gnathic-Click™ and other smartphone algorithms. *Black*: Mandibular index of Horn [24]. *Blue*: Mimouni index [53]: Length Ratios. *Red*: Camper’s angle of Sistenich [46] (see Table 2).

The application uses a hybrid algorithm that integrates five scientifically validated anthropometric indices with a random forest machine-learning model [Supplementary Material] The core logic of the system is based on anthropometric indices selected for their relevance to defining the mandibular and maxilla facial relationship as found in the craniofacial research literature. These indices provide a quantitative framework derived from specific facial landmarks (Table 2, Figure 4).

*Gnathic-Click™* uses smartphone cameras and AI to analyze facial profiles through non-invasive photogrammetry. Research shows that this method is an effective, radiation-free substitute for cephalometry when evaluating soft-tissue features [47,48,49]. The app identifies retrognathic (or prognathic) growth patterns in infants by comparing mandibular development against the maxillary and cranial base. By using stereometric based depth estimation, these applications empower users to construct 3D models and derive accurate measurements using only the device’s camera. This technology aims to transform subjective clinical observations into objective, measurable data for identifying micrognathia to encourage proactive clinical care (Table 1).

The advantage of this evolving technology to diagnose mandibular posture allows providers to recommend pacifiers with properly designed shields that allow for free mandibular protrusive and lateral movement that can encourage growth of an insufficient mandible. What could be more important than a pacifier shield that allows for proper development of the infant mandible? The Gnathic-Click application is one that can be widely adopted across the infant-care ecosystem to reach providers and caregivers at scale.

A reliable screening tool for children’s oral health that can be operated using widely available existing technology—most smartphones will do—has vast and promising potential to make non-invasive and early diagnosis infinitely more accessible than it is today. The delivery mechanism is certainly progressive and new, and continued research is critical, but it’s important to note that the concepts presented here that underpin its design and application are grounded in sound and historic study and evidence. Other references on anthropology, methodology, and applications are included here for researchers wanting to develop new photogrammetric approaches for programming the use of biometrics into smartphone photography [56,57].

## 5. Limitations

There are limitations in camera orientation, multiple image fields, minimizing distortion, and shadow correction, but most can be addressed with cellphone applications in practice environments. Other limitations owing to head positioning, lack of depth perception, and magnification errors must be considered. Future studies should integrate three-dimensional modeling applications to enhance photogrammetric accuracy and clinical applicability [58].

Ideally, some updated studies need to be done to quantify mandibular position with radiographic and 3D CAT images, but this approach is unlikely to comply with IRB approval guidelines for ethical considerations. This necessitates heavy reliance on data from early studies.

Although pacifiers are already being developed with an offset shield” to allow free movement of the mandible, we must encourage baby product development companies to participate in and offer research on their specific pacifier and shield designs that will address healthy development of the CFRC. The Sistenich 2022 paper provides an ideal for modeling a study design to evaluate shield/chin interaction [46].

It is difficult to define experience of a provider a “clinical impression”, in the absence of diagnostic imaging. Clinically, prospective studies are needed, to correlate provider “clinical impression” to algorithmic computer diagnosis using non-invasive smartphone technologies.

## 6. Conclusions

1. The pacifier shield can put retrusive pressure on the rapidly growing mandible and TMJ and prevent important free and unrestrictive movement of the mandible during NNS on a pacifier.
2. The pacifier shield plays an important role when considering the functional implications on the development of the CFRC of the baby/toddler.
3. Accurate early diagnosis, with scientific facial recognition technology embedded in smartphone camera applications, provide the data acquisition for a non-invasive screening and evaluation of retrognathic tendencies.
4. The use of Gnathic-Click app technology will build a database to help clinicians identify non-syndromic retrognathic and prognathic patients that will benefit from early diagnosis and interceptive treatment modalities.
5. Further research is needed on the evolving importance of free mandibular jaw movement on mandibular growth, the respiratory effort and the effect on the infant airway.

## Figures and Tables

**Table 1:** Diagnosis of Retrognathia with noninvasive technology advantages early intervention and education.

Topics	
Cranio-Facial Respiratory Complex (CFRC)	Educate parents and other caregivers
Airway SIDS/ALTE/BRUE/OSA	Maintain palatal width by biometric sizing of pacifiers
Airway/Sleep apnea (OSV)	Biometric shield—Free mandibular movement
Tethered Tissue	Mandibular Frenum—Breastfeeding—Tongue movement—Positioning—Free Mandibular movement
App Technology	- Pacified^®^—identify biometric appropriate pacifiers - Gnathic Click^™^—identify non syndromic phenotypes - Longitudinal follow up with objective metrics - Determination of intervention success
Early treatment orthodontics	Myofunctional therapy training/Expansion of the maxillary arch in Primary dentition—determination of palatal tongue space
Parental concerns (esthetics)	Consultations with data backed recommendations

**Table 2:** Soft tissue facial Indices in Gnathic-Click application used to predict Retrognathia.

Index Name	Calculation/Description	Relevant Soft Tissue Landmarks	Purpose/Interpretation	Reference
Horn Mandibular Index (MI)	Ratio of distance from inferior otobasion (OBI) to nasion and from OBI to gnathion	OBI, Nasion (N) Gnathion (GN)	Assesses vertical mandibular positioning. Analysis of mandibular size.	Horn et al., 2006 [24]
Mimouni Goniomaxillar/Goniomandibular Length Ratio	Ratio of distance from gonion to subnasale and from gonion to gnathion	Gonion (Go), Subnasale (Sn), Gnathion (gn)	Measures anteroposterior relationship of maxilla and mandible. Determines jaw position.	Mimouni et al., 2021 [53]
Basart Mandibular Intraoral Volume	Tetrahedral volume using labium inferius (lip), gnathion, and lower left/right otobasion (ear)	Labium Inferius, Gnathion, Left/Right OBI define tetraheron	3D photogrammetry. Estimates mandibular space volume.	Basart et al., 2018 [48]
Gamboa Ratio	Ratio of distance from tragus to subnasale and from tragus to gnathion	Tragus, Subnasale, Gnathion	Provides additional view on mandibular position	Gamboa Criado, 2016 [54]
Camper’s Angle	Angle between lines: subnasale to gonion and subnasale to tragus	Subnasale, Gonion, Tragus	Relates mandibular plane inclination to cranial base	Subtelny, 1959 [55]. Sistenich, 2022 [46]

## Data Availability

All presented data is available from the peer reviewed literature and is referenced in the manuscript.

## Supplementary Materials

The following supporting information can be downloaded at: https://www.mdpi.com/article/10.3390/children13050643/s1, Figure S1: Video Frames; Videos S1–S3: Video images licensed from Shutterstock 22 February 2026 (CS-03331-1F64).

## References

[1] Tesini D.A., Friedman C., Kethu A., Hendricks K.W. (2025). Pacifier sizing as a prescription for better oral health outcomes for infants: A call to action. Children.

[2] Liu Y.-P., Behrents R.G., Buschang P.H. (2010). Mandibular growth, remodeling, and maturation during infancy and early childhood. Angle Orthod..

[3] Schipper J.A.M., van Lieshout M.J.S., Böhringer S., Padwa B.L., Robben S.G.F., van Rijn R.R., Koudstaal M.J., Lequin M.H., Wolvius E.B. (2021). Modelling growth curves of the normal infant’s mandible: 3D measurements using computed tomography. Clin. Oral Investig..

[4] Lipowicz A., Kawlewska E., Larysz D., Kostyra P., Graja K., Joszko K., Gzik-Zroska B., Wolański W. (2023). Evaluation of mandibular geometry in healthy children aged 0–1 year—A pilot study. Acta Bioeng. Biomech..

[5] Iwayama K., Eishima M. (1997). Neonatal sucking behaviour and its development until 14 months. Early Hum. Dev..

[6] Roberts W.E., Goodacre C.J. (2020). The temporomandibular joint: A critical review of life-support functions, development, articular surfaces, biomechanics and degeneration. J. Prosthodont..

[7] Nakai H., Niimi A., Ueda M. (1998). The influence of compressive loading on growth of cartilage of the mandibular condyle in vitro. Arch. Oral Biol..

[8] Nickel J.C., McLachlan K.R., Smith D.M. (1988). A Theoretical model of loading and eminence development of the postnatal human temporomandibular joint. J. Dent. Res..

[9] Matsubara M., Inoue M. (2019). A comparison of the movement of the mandible in infants between breastfeeding and bottle-feeding. J. Nurs. Sci. Eng..

[10] Moral A., Bolibar I., Seguranyes G., Ustrell J.M., Sebastiá G., Martínez-Barba C., Ríos J. (2010). Mechanics of sucking: Comparison between bottle feeding and breastfeeding. BMC Pediatr..

[11] Endo Y., Mizutani H., Yasue K., Senga K., Ueda M. (1998). Influence of food consistency and dental extractions on the rat mandibular condyle: A mor-phological, histological and immunohistochemical study. J. Cranio-Maxillofac. Surg..

[12] Engelsma S.O., Jansen H.W.B., Duterloo H.S. (1980). An invivo transplantation study of growth of the mandibular condyle in a functional position in the rat. Arch. Oral Biol..

[13] Duterloo H.S. (1967). ‘In vivo’ Implantation of the Mandibular Condyle of the Rat: An Experimental Investigation of the Growth of the Lower Jaw. Ph.D. Thesis.

[14] Tonkin S. (1975). Sudden infant death syndrome: Hypothesis of causation. Pediatrics.

[15] Tonkin S.L., Vogel S., Bennet L., Gunn A.J. (2002). Positional upper airways narrowing and an apparent life threatening event. N. Z. Med. J..

[16] Reed W.R., Roberts J.L., Thach B.T. (1985). Factors influencing regional patency and configuration of the human infant upper airway. J. Appl. Physiol..

[17] Kim J.C., Yang K.H., Lee K.S. (1993). The effect of retractive force on jaw growth in growing rabbits. Korean J. Orthod..

[18] Cozzi F., Albani R., Cardi E. (1979). A common pathophysiology for sudden cot death and sleep apnoea. “The vacuum-glossoptosis syndrome”. Med. Hypotheses.

[19] Tonkin S.L., Vogel S.A., Gunn A.J. (2007). Upper airway size while sucking on a pacifier in an infant with micrognathia. J. Paediatr. Child Health.

[20] Kikuchi Y. (2008). Three-dimensional relationship between pharyngeal airway and maxillo-facial morphology. Bull. Tokyo Dent. Coll..

[21] Huang Y.-S., Guilleminault C. (2013). Pediatric obstructive sleep apnea and the critical role of oral-facial growth: Evidences. Front. Neurol..

[22] Gunn T.R., Tonkin S.L., Hadden W., Davis S.L., Gunn A.J. (2000). Neonatal micrognathia is associated with small upper airways on radiographic measurement. Acta Paediatr..

[23] Tonkin S.L., Lui D., McIntosh C.G., Rowley S., Knight D.B., Gunn A.J. (2007). Effect of pacifier use on mandibular position in preterm infants. Acta Paediatr..

[24] Horn M.H., Kinnamon D.D., Ferraro N., Curley M.A. (2006). Smaller mandibular size in infants with a history of an apparent life-threatening event. J. Pediatr..

[25] Abed B.Z., Oneto S., Abreu A.R., Chediak A.D. (2020). How might non nutritional sucking protect from sudden infant death syndrome. Med. Hypotheses.

[26] Fleming P.J., Blair P.S., Pollard K., Platt M.W., Leach C., Smith I., Berry P.J., Golding J., The CESDI SUDI Research Team (1999). Pacifier use and sudden infant death syndrome: Results from the CESDI/SUDI case control study. Arch. Dis. Child..

[27] Cozzi F., Morini F., Tozzi C., Bonci E., Cozzi D.A. (2002). Effect of pacifier use on oral breathing in healthy newborn infants. Pediatr. Pulmonol..

[28] Levrini L., Riccaboni F., Maurino V., Azzi L., Nosetti L. (2021). Effects of pacifiers on peripheral capillary oxygen saturation during wake time. Appl. Sci..

[29] Martinot J.-B., Le-Dong N.-N., Cuthbert V., Denison S., Silkoff P.E., Guénard H., Gozal D., Pépin J.L., Borel J.C. (2017). Mandibular Movements As Accurate Reporters of Respiratory Effort During Sleep: Validation Against Diaphragmatic Electromyography. Front. Neurol..

[30] Martinot J.-B., Le-Dong N.-N., Cassibba J., Clause D., Pépin J.-L., Gozal D. (2025). Interpreting the mandibular jaw movement signal in pediatric obstructive sleep apnea diagnosis: A technical and practical review. Sleep Med. Rev..

[31] Sharma A. (2025). Pacifier Dilemma: Benefits, Risks and Best Practices for Your Child.

[32] Renz-Polster H., Blair P.S., Ball H.L., Jenni O.G., De Bock F. (2024). Death from failed protection? An evolutionary-developmental theory of sudden infant death syndrome. Hum. Nat..

[33] Li D.-K., Willinger M., Petitti D.B., Odouli R., Liu L., Hoffman H.J. (2005). Use of a dummy (pacifier) during sleep and risk of sudden infant death syndrome (SIDS): Population based case-control study. BMJ.

[34] Cohen M., Brown D.R., Myers M.M. (2001). Cardiovascular responses to pacifier experience and feeding in newborn infants. Dev. Psychobiol. J. Int. Soc. Dev. Psychobiol..

[35] Horne R.S., Hauck F.R., Moon R.Y., L’hoir M.P., Blair P.S., Physiology and Epidemiology Working Groups of the International Society for the Study and Prevention of Perinatal and Infant Death (2014). Dummy (pacifier) use and sudden infant death syndrome: Potential advantages and disadvantages. J. Paediatr. Child Health.

[36] Hanzer M., Zotter H., Sauseng W., Pfurtscheller K., Müller W., Kerbl R. (2009). Pacifier use does not alter the frequency or duration of spontaneous arousals in sleeping infants. Sleep Med..

[37] Weiss P., Kerbl R. (2001). The relatively short duration that a child retains a pacifier in the mouth during sleep: Implications for sudden infant death syndrome. Eur. J. Pediatr..

[38] Colley J.R.T., Creamer B. (1958). Sucking and Swallowing in Infants. BMJ.

[39] Anderson G.C., McBride M.R., Dahm J., Ellis M.K., Vidyasagar D. (1982). Development of sucking in term infants from birth to four hours postbirth. Res. Nurs. Health.

[40] Lindner A. (1991). Measurement of intra-oral negative air pressure during dummy sucking in human newborn. Eur. J. Orthod..

[41] Lindner A., Hellsing E. (1991). Cheek and lip pressure against maxillary dental arch during dummy sucking. Eur. J. Orthod..

[42] Asano T. (1986). The effects of mandibular retractive force on the growing rat mandible. Am. J. Orthod. Dentofac. Orthop..

[43] Muñoz L.E., Kartushina N., Mayor J. (2024). Sustained pacifier use is associated with smaller vocabulary sizes at 1 and 2 years of age: A cross-sectional study. Dev. Sci..

[44] Stüdeli T. (2014). Ergonomic recommendations for the design of pacifiers. Advances in Ergonomics in Design, Usability, & Special Populations: Part II. Open Access Science in Human Factors Engineering and Human Centered Computing.

[45] Levrini L., Paracchini L., Ricci L., Sparaco M., Saran S., Mulè G. (2025). Biomechanical Analysis of Different Pacifiers and Their Effects on the Upper Jaw and Tongue. Appl. Sci..

[46] Sistenich G., Middelberg C., Stamm T., Dirksen D., Hohoff A. (2022). Conformity between pacifier design and palate shape in preterm and term infants considering age-specific palate size, facial profile and lip thickness. Children.

[47] Nucera R., Lo Giudice A., Bellocchio M., Spinuzza P., Caprioglio A., Cordasco G. (2017). Diagnostic concordance between skeletal cephalometrics, radiograph-based soft-tissue cephalometrics, and photograph-based soft-tissue cephalometrics. Eur. J. Orthod..

[48] Basart H., Suttie M.M., Ibrahim A., Ferretti P., van der Horst C.M., Hennekam R.C., Hammond P. (2018). Objectifying micrognathia using three-dimensional photogrammetric analysis. J. Craniofac. Surg..

[49] Jaiswal P., Gandhi A., Gupta A.R., Malik N., Singh S.K., Ramesh K. (2021). Reliability of Photogrammetric Landmarks to the Conventional Cephalogram for Analyzing Soft-Tissue Landmarks in Orthodontics. J. Pharm. Bioallied Sci..

[50] Arshad F., Prashanth C.S., Amarnath B.C. (2024). Accuracy of Facial Soft Tissue Measurements Using Photographic Analysis Method. RGUHS J. Dent. Sci..

[51] Caple J., Stephan C.N. (2015). A standardized nomenclature for craniofacial and facial anthropometry. Int. J. Leg. Med..

[52] Blahnik V., Schindelbeck O. (2021). Smartphone imaging technology and its applications. Adv. Opt. Technol..

[53] Mimouni G., Merlob P., Mimouni F.B., Bin-Nun A. (2020). The goniomaxillar length/goniomandibular length ratio in normal newborn infants: A clinical tool for defining chin position abnormalities. Am. J. Med. Genet. Part A.

[54] Gamboa Criado Y.A. (2016). Antropometria General y Craniofacial en Neonatos. Caso Bogotá Durante Los Años 2011–2014. Ph.D. Thesis.

[55] Subtelny J.D. (1959). A longitudinal study of soft tissue facial structures and their profile characteristics, defined in relation to underlying skeletal structures. Am. J. Orthod..

[56] Andrews J., Alwafi A., Bichu Y.M., Pliska B.T., Mostafa N., Zou B. (2023). Validation of three-dimensional facial imaging captured with smartphone-based photogrammetry application in comparison to stereophotogrammetry system. Heliyon.

[57] Dianat I., Molenbroek J., Castellucci H.I. (2018). A review of the methodology and applications of anthropometry in ergonomics and product design. Ergonomics.

[58] Fatima F., Hallolli C., Tubaki R., Shah I.F., Thekiya A.H., Tabassum H., Gupta S. (2025). Comparative Evaluation of Photogrammetric, Radiographic, and Direct Measurements in Facial Analysis: A Cross-Sectional Study. Cureus.

