# Supplementary material for: The Role of a Pacifier Shield: A Unique Perspective on Mandibular Growth and Airway Health: Part 2
Source: Children (Basel). 2026 May 4;13(5):643. doi: 10.3390/children13050643 (PMC13204359; doi:10.3390/children13050643)
Supplement: Supplementary file 1 [file children-13-00643-s001.zip › children-4250237-supplementary.pdf]

<u>Supplemental Material:</u>
The Role of a Pacifier Shield: A Unique Perspective on Mandibular Growth and Airway Health: Part 2.

<u>The Functional Pacifier Shield</u>

The movement of the pacifier is caused by the peristaltic movement of the tongue and periodicity of the intraoral sucking pressure against the bulb nipple. Additional impact of the compartmentalization of negative intraoral pressures around the bulb, between the tongue, palate and bulb serve to cause forward movement of the bulb into the palate and tipping of the pacifier shield into the chin and mandible. Mostly all pacifier shields are flat the impact of the force against the chin is proportional to the movement of the upper (top) part of the shield away from the face.
for

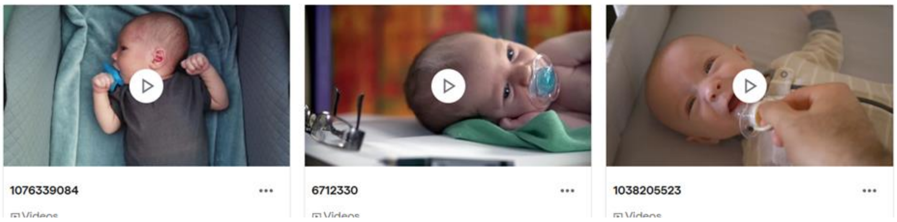

(a) (b) (c).

Figure S1: Three video images of infants sucking on a pacifier. *.

(a) Direct pressure localized to chin from large shield (b) slow motion of shield contact against mandible and chin. (c) rapid sucking bursts showing frequent impact on the mandibular alveolar ridge at the mentolabial sulcus (mental crease).

(a) Direct pressure localized to chin with additional stabilization from baby's hand creating force.
(b) In this slow motion video you will notice the motion of the top portion of the pacifier shield moving away from the maxillary area of the face. Because the shield of the pacifier, is flat, the lower (bottom portion) of the shield impinges on the jaw in the area of the chin. This limits movement of the chin downward and inward.
(c) After this fussy baby is offered a pacifier, he begins to forcefully suck creating the "rocking" motion of the pacifier shield, away (top of shield) and into the chin (bottom of shield). This creates frequent pressure challenges limiting free mandibular movement.

Future FEA (Finite Element Analysis) and other advanced engineering models will evaluate the contact pressures calculated as force per unit area of forces on the chin. This will allow prediction of shield pressure dependent on facial type, shield size, shield design, angle of bulb to shield and depth of palate.

- Video images licensed from Shutterstock Feb 22, 2026 (CS-03331-1F64).

<u>The Evolving Technology for Predictive Non-invasive Diagnosis</u>

Gnathic-Click™ offers a transformative approach by integrating established scientific knowledge with cutting-edge technology.

The core logic of the Gnathic-Click™ application is rooted in scientifically validated anthropometric indices selected based on their relevance to the evaluation of the mandibular and maxillofacial relationships as documented in the craniofacial research literature (Table 2). These indices provide a quantitative framework derived from specific facial landmarks.

The application employs a sophisticated algorithm built upon Facial Recognition and a Random Forest Algorithm. This is a powerful ensemble learning method used for classification. It operates by constructing a multitude of decision trees during training. For a new input (in this case, the derived anthropometric measurements and age), each tree provides a classification, and the forest outputs the class that is the mode (most frequent prediction) of the individual trees. Data to define a Reference Standard can be compiled by an experienced clinician to provide a *clinical impression* (Retrognathic, Orthognathic, or Prognathic). This approach is robust against overfitting and generally yields high accuracy.

Developmental Staging and Clinical Validation

It is important to clarify the current developmental staging of this diagnostic technology. The initial algorithm training and preliminary framework described herein represents a pre-validation description of proof-of-concept. During this stage, the foundational logic and reference standards of the application were established utilizing retrospective data and pediatric dentists' expert *clinical impression*. This stage focused on technological modeling rather than on prospective human subject research.-Pre-Validation serves strictly as a technological baseline to demonstrate the viability of integrating established anthropometric indices with machine learning classification.

To transition from this proof-of-concept to a validated clinical tool, a rigorous "Phase 1" clinical validation is planned.-Phase 1 will systematically evaluate the Gnathic-Click™ algorithmic predictions against definitive, accepted diagnostic imaging standards across a diverse pediatric cohort. This formalized clinical validation is an essential next step to confirm the application's diagnostic accuracy, reliability, and safety for widespread integration into early pediatric screening.
